# Exploring the role of brain-derived extracellular vesicles in viral infections: from pathological insights to biomarker potential

**DOI:** 10.3389/fcimb.2024.1423394

**Published:** 2024-06-03

**Authors:** Larise Oberholster, Renaud Du Pasquier, Amandine Mathias

**Affiliations:** ^1^ Laboratory of Neuroimmunology, Service of Neurology, Department of Clinical Neurosciences, Lausanne University Hospital (CHUV) and University of Lausanne, Lausanne, Switzerland; ^2^ Service of Neurology, Department of Clinical Neurosciences, Lausanne University Hospital (CHUV) and University of Lausanne, Lausanne, Switzerland

**Keywords:** extracellular vesicles, neurotropic viruses, biomarker, pathogenesis, herpes simplex, polyomavirus, Zika virus, HIV

## Abstract

Extracellular vesicles (EVs) are membrane-bound vesicles secreted by all cell types that play a central role in cell-to-cell communication. Since these vesicles serve as vehicles of cellular content (nucleic acids, proteins and lipids) with the potential to cross biological barriers, they represent a novel attractive window into an otherwise inaccessible organ, such as the brain. The composition of EVs is cell-type specific and mirrors the physiological condition of the cell-of-origin. Consequently, during viral infection, EVs undergo significant changes in their content and morphology, thereby reflecting alterations in the cellular state. Here, we briefly summarize the potential of brain-derived EVs as a lens into viral infection in the central nervous system, thereby: 1) uncovering underlying pathophysiological processes at play and 2) serving as liquid biopsies of the brain, representing a non-invasive source of biomarkers for monitoring disease activity. Although translating the potential of EVs from research to diagnosis poses complexities, characterizing brain-derived EVs in the context of viral infections holds promise to enhance diagnostic and therapeutic strategies, offering new avenues for managing infectious neurological diseases.

## 1 Introduction

Extracellular vesicles (EVs) are membranous structures that are secreted by all cell types and that are found in all body fluids, as well as tissue culture supernatants (van Niel et al., 2018). While the term `extracellular vesicles` is used to annotate all cell-derived membrane-delimited vesicles, these structures are highly divergent and can be divided into two main groups, namely exosomes and microvesicles, that are characterized based on their biogenesis, content, morphology, and functional atributes (van Niel et al., 2018). Exosomes, referred to as small EVs, are the most widely studied and comprise a size distribution ranging from 30 nm to 150 nm (Johnstone et al., 1987). Exosomes arise from the inward budding of early endosomes that enables formation of intraluminal vesicles (ILVs) within multivesicular endosomes (MVEs) (Harding et al., 1984). MVEs can fuse with the plasma membrane, releasing the ILVs, now referred to as exosomes, into the extracellular space (Pan et al., 1985; Doyle and Wang, 2019). Contrary to exosomes, microvesicles bud directly from the plasma membrane into the extracellular environment and have a size distribution ranging from 100 nm to 1000 nm (Tricarico et al., 2017). While, historically characterized as means of eliminating cellular waste (Johnstone et al., 1987), it is now established that EVs play a critical role in intercellular communication, transporting cellular components such as microRNAs, mRNAs, and DNAs, lipids and proteins, in both physiological and diseased conditions (Colombo et al., 2014; Lo Cicero et al., 2015; Yanez-Mo et al., 2015; van Niel et al., 2018). The transfer of these molecules via EVs can have a range of effects on recipient cells, including alteration of gene expression, modulation of signaling pathways, induction of phenotypic changes, and influence on the cellular behavior (Raposo et al., 1996; Valadi et al., 2007; van Niel et al., 2018). The composition of EVs is cell type-specific and determined by the physiological state of the cell, along with environmental stimuli (Minciacchi et al., 2015). Here, we compile evidence for the potential of EVs to enhance our understanding of the brain and its associated disease mechanisms, particularly in the context of viral infections. Additionally, we will briefly review the challenging potential of EVs as new disease biomarkers.

## 2 EVs from health to infection

### 2.1 EVs in the brain during physiological conditions

The central nervous system (CNS), being comprised of highly divergent populations of neuronal or glial cells generated in a spatiotemporal manner, stands as one of the most intricate structures in the body. The importance of EVs in intercellular communication in the CNS has been extensively characterized (Fruhbeis et al., 2012; Kramer-Albers and Hill, 2016). EVs might have a central role in neural development (Edlund and Jessell, 1999; Bahram Sangani et al., 2021) by harboring neurodevelopmental signaling proteins with the capacity to induce neural progenitor proliferation and differentiation as demonstrated *in vitro* and *in vivo* in developing mouse neural models (Sharma et al., 2019). Moreover, EVs were also found to have neuroprotective function(s) by assisting in the clearance of β-amyloid from the CNS *in vivo* (Yuyama et al., 2014), or to protect human brain endothelial cells from oxidative stress as shown *in vitro* (Liu et al., 2017). EVs also have a role in maintaining and enhancing synaptic plasticity (Graykowski et al., 2020). Neuron-derived EVs were found to contain synaptic proteins and upon internalization by cultured neurons, promoted dendritic spine formation through BDNF-TrkB-mediated signaling. Moreover, these neuron-derived EVs were able to preserve neuronal complexity during nutrient deprivation (Solana-Balaguer et al., 2023).

### 2.2 EVs and viral infection

Besides their involvement in normal physiological conditions in the brain, EVs have also been implicated in mediating a pathological effect on recipient cells. Indeed, mounting evidence suggests a close link between EVs and viruses, as viruses often rely on the same cellular machinery involved in EV biogenesis for their own replication and release into the extracellular milieu (Bello-Morales et al., 2020). Viruses may even utilize EVs as a means of propagation to neighboring cells (Moulin et al., 2023). Viruses typically categorized as non-enveloped, such as hepatitis A virus (HAV) (Feng et al., 2013), Coxsackievirus (Robinson et al., 2014), rotavirus and norovirus (Santiana et al., 2018) have been demonstrated to be enclosed within membrane vesicles when secreted by cells. The encapsulation of multiple virions/genomes within a single EV allows for the delivery of a higher multiplicity of infection, thereby increasing the possibility of productive infection and enabling the replacement of faulty proteins or particles (Troyer and Tilton, 2021). Moreover, the physical encapsulation by EVs also offers protection against neutralizing antibodies as has been reported for herpes simplex virus 1 (HSV-1) (Heilingloh et al., 2015; Bello-Morales et al., 2018), HAV (Feng et al., 2013), Hepatitis C virus (HCV) (Ramakrishnaiah et al., 2013), and Hepatitis E virus (HEV) (Nagashima et al., 2014).

On the other hand, EVs play a crucial role in regulating viral infections by serving as carriers of viral components or immunomodulatory molecules that can have either a pro- or anti-viral effect on recipient cells (Perez et al., 2019). For instance, EVs released by HIV-1 infected primary CD4+ T lymphocytes were shown to deliver negative factor protein (Nef) to nearby cells, licensing them for viral infection (Arenaccio et al., 2014). Conversely, EVs can also serve as carriers of anti-viral mediators such as human cytidine deaminase APOBEC3G, known for its role in cellular defense against retrovirus (Khatua et al., 2009) or, stimulator of interferon genes (STING), which can activate the innate immune response against viruses like HSV-1, limiting viral spread (Deschamps and Kalamvoki, 2018). Similarly, EVs containing viral nucleic acids, such as HCV RNA (Dreux et al., 2012) or Epstein-Barr virus (EBV) polymerase III-transcribed noncoding RNAs (EBERs) (Baglio et al., 2016) can induce an innate immune response in recipient cells.

Understanding how EVs facilitate or hinder viral entry, replication or spread relies heavily on the precise isolation of these two populations. However, due to their similar size and buoyant density, separating EVs from free virions presents a significant hurdle (Arakelyan et al., 2017; Kutchy et al., 2020). Techniques for separation include targeting EV specific membrane proteins (CD63, CD9, CD81) on the surface of EVs (Thery et al., 2018; Welsh et al., 2024), or exploiting the migration differences between EVs and viruses using Iodixanol velocity gradient (Konadu et al., 2016). Nevertheless, assessing EV purity from virus-infected cells is challenging as the border between EVs and some enveloped viruses remains ill-defined (Nolte-’t Hoen et al., 2016). Nonetheless, new technics such as high-throughput flow cytometry may enable future discrimination of EV and virus particles (Nolte-’t Hoen et al., 2012; Arakelyan et al., 2015).

## 3 Neurotropic viruses and EV hijacking: example of brain infections

Examining EVs in the context of viral infection might shed light into disease mechanisms, particularly in inaccessible organs such as brain. Here, we focus on selected viruses that can invade the CNS and the role of EVs in their interaction with CNS target cells.

### 3.1 Flaviviruses

The unique evolutionary advantages offered by EVs have also been harnessed by neurotrophic viruses belonging to the family of *Flaviviridae.* This includes Zika virus (ZIKV), which have received major attention for its propensity to evoke serious neurological injury during the early stages of human development (Brasil et al., 2016). Furthermore, major progress has been achieved using mouse and non-human primate models, revealing that ZIKV circumvents host cellular machinery through a mechanism referred to as the “secretory autophagy” or “exosome pathway” process that may facilitate viral vertical transmission from trophoblast to fetal cells during pregnancy (Cugola et al., 2016; Zhang et al., 2017; Wu et al., 2023). To further elucidate the role of EVs in ZIKV infection within the developing fetal brain, EVs from cultured ZIKV-infected mouse neurons were analyzed for viral content and potential to mediate viral infection to uninfected cortical neurons (Zhou et al., 2019). EVs from infected cells were shown to comprise ZIKV RNA and proteins that were highly infectious and resistant to RNase activity and antibody neutralization treatment. In the same study, ZIKV was shown to induce both the activity and gene expression of neutral sphingomyelinase SMPD3 (nSMase2) in cortical neurons. This induction facilitated infection and transmission of ZIKV through EVs, potentially leading to severe neuronal death. Such neuronal damage may result in neurological manifestations, such as microcephaly, in the developing embryonic brains (Zhou et al., 2019). Similar observations were done on EVs derived from Japanese Encephalitis virus (JEV) -infected microglial cells which, upon internalization by neurons, induced caspase activation and neuronal injury (Mukherjee et al., 2019).

### 3.2 Herpesviruses

HSV-1, a prevalent neurotropic virus, typically infects sensory neurons, traveling to trigeminal ganglia through retrograde axonal transport (Marcocci et al., 2020; Zhu and Viejo-Borbolla, 2021). In rare cases, the virus can infect the brain parenchyma, particularly the temporal lobes, causing a herpes simplex meningo-encephalitis (Marcocci et al., 2020). EVs from HSV-1-infected human oligodendroglial HOG cells were shown to propagate viral infection to Chinese hamster ovary (CHO) cells, which are normally resistant to free virions, suggesting a role for EVs in broadening HSV-1 tropism and viral immune evasion (Bello-Morales et al., 2018). More recently, EVs from HSV-1-infected epithelial cells were demonstrated to exhibit an upregulation of proteins with the capacity to promote neurite outgrowth and subsequent viral spread to neurons (Sun et al., 2024).

### 3.3 Human Immunodeficiency Virus (HIV)

EVs might also contribute to the neurotoxicity associated with cognitive impairment in patients suffering from acquired immunodeficiency syndrome. In a study investigating the impact of opiate drug abuse on the progression of HIV-associated neurocognitive disorder, EVs derived from astrocytes treated with HIV tat protein and morphine was shown to contain an increased level of miR-29b. Upon uptake by human neuronal cell lines in culture, this heightened miR-29b content led to neuronal death through the targeting and downregulation of platelet-derived growth factor-B (PDGFB) expression (Hu et al., 2012). Furthermore, EVs from HIV tat-exposed primary human fetal astrocytes contained increased levels of miR-7 that induced synaptic injury through the downregulation of neuronal neuroligin 2 (NLGN2) in recipient neurons (Hu et al., 2020). In a separate study, HIV Nef protein was found to associate with microglia-derived EVs and, using an *in vitro* model of the blood-brain barrier (BBB), was shown to compromise the permeability and integrity of the BBB (Raymond et al., 2016). Moreover, these Nef-containing EVs triggered the upregulation of Toll-like receptor-induced cytokines and chemokines, suggesting a role of Nef-containing vesicles in neuroinflammation and subsequent CNS injury observed during HIV-associated neurocognitive disorder (HAND) (Raymond et al., 2016). Additionally, exposure of neurons to Nef-containing astrocyte EVs resulted in the suppression of neuronal action potential and induction of oxidative stress and neurotoxicity (Sami Saribas et al., 2017).

### 3.4 Polyomaviruses

Recent studies have focused on the involvement of EVs in the dissemination of JC polyomavirus (JCPyV) in the CNS. JCPyV is the causative agent of progressive multifocal leukoencephalopathy, a severe demyelinating disease of the CNS that results in the formation of lesions across the brain parenchyma (Padgett et al., 1971; Cortese et al., 2021). We have recently shown that JCPyV alters the proteomic content of EVs from infected human induced pluripotent stem-cell derived astrocytes, which resulted in a signature highly reflective of infected cells, yet starkly different from that of EVs generated under inflammatory, non-viral, conditions (Oberholster et al., 2023). The involvement of EVs in the *in vitro* propagation of JCPyV has been demonstrated in different cell types, suggesting an alternative route of dissemination beyond the conventional release of free virus particles (Morris-Love et al., 2019; Morris-Love et al., 2022). Moreover, it was demonstrated that EVs from JCPyV-infected choroid plexus epithelial cells were able to transmit viral infection to naïve glial cells that lacked the alleged viral attachement receptor, namely lactoseries tetrasaccharide c (O’Hara et al., 2020). These data suggest that JCPyV infection could be, at least partially, independent of the virus attachment receptor. Furthermore, JCPyV VP1 capsid protein and genomic DNA of the archetype sequence was found to associate with plasma derived EVs from HIV patients, suggesting a potential role for EVs in JCPyV spread *in vivo* and entry into the CNS (Scribano et al., 2020). However, due to limitations in modeling JCPyV infection in the brain, the significance of EVs in the dissemination of JCPyV within the CNS remains unclear.

Interestingly, since the contents of EVs from infected or healthy cells differ profoundly in their composition (Meckes et al., 2013; Oberholster et al., 2023), EVs offer a promising avenue for understanding the complex mechanisms at play in the context of viral infection in the brain. This, in turn, might pave the way toward the development of innovative diagnostic tools and therapeutic interventions targeting neurological diseases.

## 4 EVs as a source of biomarkers for neurological diseases

Identifying biomarkers for neurological diseases, including those that result from viral infections, is especially challenging as most neurodegenerative disorders are confined to 1) an inaccessible organ, 2) a distinct region of the brain and 3) a particular subset of cells. However, various CNS cell types have been demonstrated to secrete EVs, including oligodendrocytes, neurons and astrocytes (Bakhti et al., 2011; Wu et al., 2017), suggesting that brain-derived EVs in body fluids could offer a non-invasive means of obtaining a representation of the entire brain and sustain new biomarker characterization.

Indeed, EVs offer multiple advantages as a unique source of new biomarkers due to their specificity, stability, ability to cross biological barriers and non-invasive sampling. Because the contents of EVs are shielded from degradation by DNases, RNases, and proteases, the characterization of EVs isolated from any biofluid allows for the identification of robust biomarkers, as opposed to the analysis of plain blood, urine, or CSF (Boukouris and Mathivanan, 2015).

The potential of EVs to cross biological barriers, including the BBB, has opened avenues in which CNS-derived EVs found in the CSF and in the blood could serve as non-invasive means to gain valuable insights into the physiological state of the brain (Ramos-Zaldivar et al., 2022). In patients with Parkinson’s disease (PD), multiple system atrophy (MSA) or progressive supranuclear palsy (PSP), plasma levels of oligodendrocyte- and neuron-derived EVs were shown to be increased compared to disease control group (Ohmichi et al., 2019). In AD patients, as compared to patients with frontotemporal dementia, neuron-derived blood exosomes were found to have higher levels of lysosome-associated membrane protein 1 (LAMP1) and cathepsin D (Goetzl et al., 2015). Another study found that astrocyte-derived EVs contained elevated levels of classical and alternative complement pathway proteins in AD patients as compared to matched controls (Goetzl et al., 2018). Furthermore, the analysis of EVs might enable the enrichment of low abundant proteins that are normally masked by high abundant ones, such as albumin, in crude biofluids (Upadhya et al., 2020). Yet, protein composition within EVs can be influenced by various factors, such as sample handling and storage conditions, necessitating standardized isolation and detection methods.

Among other cargos, nucleic acids encapsulated within EVs, including microRNAs (miRNAs), or messenger RNAs (mRNAs), and DNA fragments, have been demonstrated to reflect disease-specific molecular signatures, facilitating early detection and monitoring of disease progression such as mainly reported in the context of neurodegeneration (Raghav et al., 2022; Lim et al., 2023) or primary CNS tumors (Zhang et al., 2023; Hallal et al., 2024). Despite being an attractive source of EV-associated biomarkers, accurate EV-RNA/DNA quantitation faces key challenges due to low yields, influenced by sample source and isolation methods, further impacting data interpretation (Hill et al., 2013; Saugstad et al., 2017; Gandham et al., 2020). Overcoming these hurdles will be essential for reliable EV RNA/DNA characterization, particularly in disease diagnosis and therapeutic development.

Lipids associated with EVs, even if less studied to date especially as potential biomarkers, also may play pivotal roles in cellular signaling and neurotransmission within the CNS. Alterations in lipid profiles of CNS EVs may signify cellular dysfunction and disease progression, offering novel avenues for biomarker discovery such as envisioned in PD or AD (Vanherle et al., 2020). However, comprehensive lipidomic analysis of EVs requires specialized equipment and expertise further hindering transfer into routine laboratory testing as diagnosis or prognostic markers.

Another challenge in approaching CNS specific biomarkers is establishing tissue-specific EV isolation technics. To isolate brain-derived EVs from the blood, current strategies are typically based on immunoprecipitation techniques that utilize antibodies targeting cell-type specific markers: oligodendrocyte-myelin glycoprotein (MOG) for oligodendrocytes, neural cell adhesion molecule L1 (L1CAM) for neurons, and glutamate aspartate transport protein (GLAST) for astrocytes. However, the reliability of these markers for isolating ‘brain-derived’ EVs has been questioned, as these markers are also widely expressed by cells outside the CNS (Kramer-Albers and Hill, 2016). Furthermore, *in vitro* studies have suggested that these markers may not consistently be incorporated into EVs (Norman et al., 2021; You et al., 2022). Nonetheless, since brain-derived EVs in the periphery might present a real-time source of information relevant to non-accessible organ such as the brain, efforts are underway to identify new brain cell-type specific membrane makers. Research about EVs as a diagnostic tool for viral infectious diseases is still in its early stages, yet it holds significant promise. CSF EVs derived from glial cells, neurons and the choroid plexus of patients with HIV-associated neurocognitive disorders were shown to comprise proteins involved in stress responses and immune/inflammatory responses, suggesting their potential as a source of biomarkers to neurological disease activity (Guha et al., 2019). In another study, plasma neuron-derived EVs from HIV-positive patients with neurological complications showed an enrichment of neurofilament-light chain (NFL), high mobility group box 1 (HMGB1) and amyloid beta (Aβ) proteins as compared to controls (Sun et al., 2017). More recently, neuron-derived EVs isolated from patients experiencing long term effects of coronavirus disease 2019 (COVID-19) were found to contain elevated levels of proteins indicative of neuronal damage as compared to pre-COVID-19 controls (Tang et al., 2024). As such, neuron-derived EVs might provide a non-invasive source of biomarkers to identify cognitive impairment related to HIV-infection. Similarly, plasma-derived EVs expressing the astrocytic marker, glial fibrillary acidic protein (GFAP), were significantly increased in patients suffering from HAND as compared to patients with normal cognition, further supporting the relevance of brain-derived EVs as indicators of neuronal injury (de Menezes et al., 2022).

Finally, the translation of EV research into clinical applications is a concern, particularly regarding their potential as a novel source of biomarkers. Indeed, before EVs can be widely accepted as disease biomarkers, it is imperative to overcome the technical challenges posed by their isolation, characterization, methodological standardization, and reporting (Van Deun et al., 2017; Roux et al., 2020; Welsh et al., 2024). The chosen method for isolating EVs significantly affects both sample yield and purity. Currently, there is no unified standard for exosome separation and purification. Indeed, it exists various methods including differential ultracentrifugation (UC), density gradient ultracentrifugation (DGUC), size exclusion chromatography (SEC), filtration, precipitation or immune-based extraction or more recently microfluidic approaches; each of those have their advantages and limitations (Xu et al., 2016; He et al., 2022). Technical challenges in isolating EVs consistently, along with complex analysis techniques, may hinder their use as powerful biomarkers. Pre- and post-analytical variability can also impact the presence of non-vesicular particles together with the surface and content of specific EVs (Clayton et al., 2019; Buzas, 2022; Görgens et al., 2022). Prioritizing reproducibility and ease-of-use is crucial for effective biomarker discovery in any diseases. All these challenges are currently addressed. Guidelines and position papers have been recently published for blood and CSF (Hendrix, 2021; Lucien et al., 2023; Sandau et al., 2024). Resolving these challenges is essential for harnessing the full diagnostic potential of EVs in clinical settings.

## 5 Discussion

**Figure 1:**
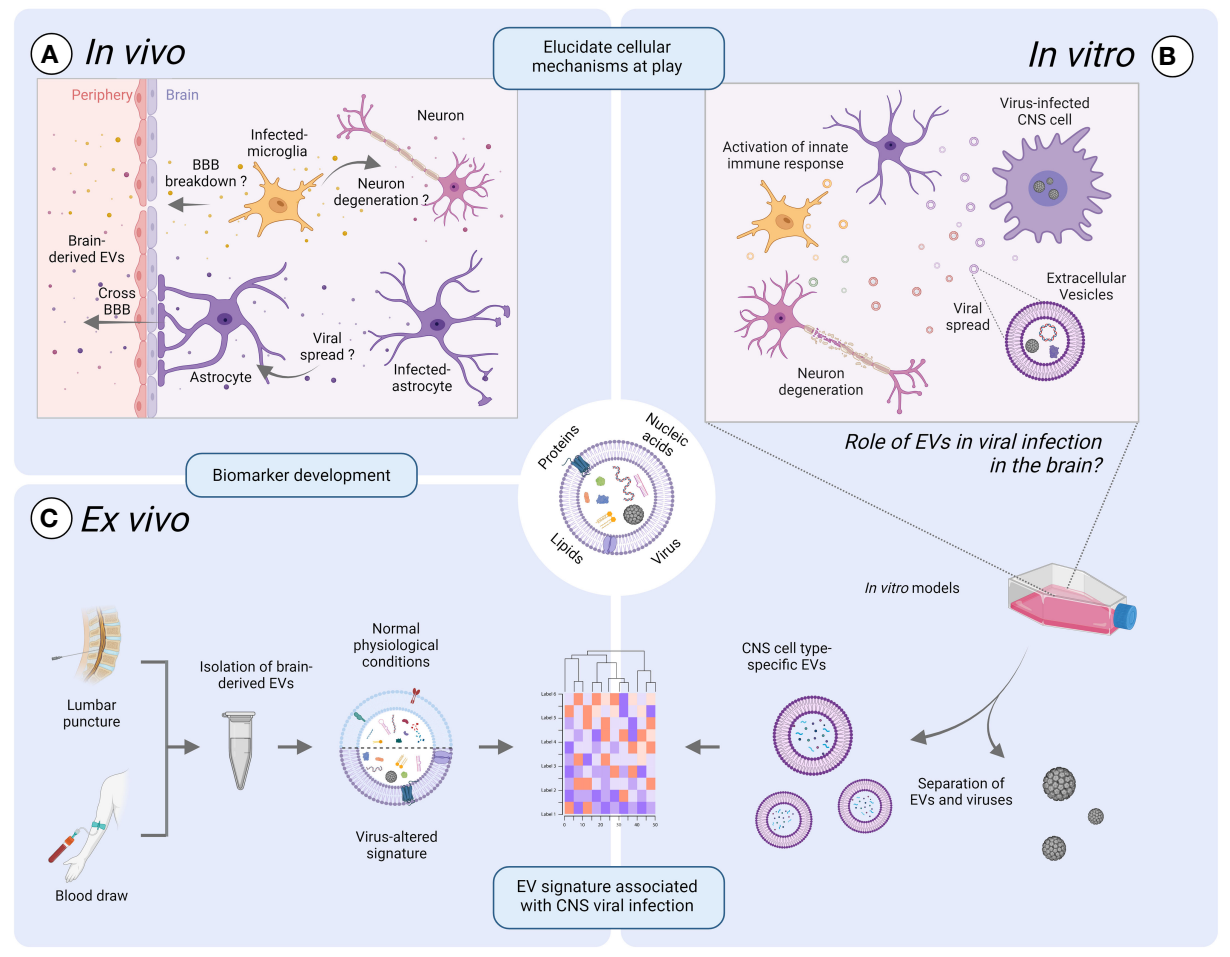
Exploring the promise of extracellular vesicles (EVs) in viral infection of the brain. EVs might play a vital role in uncovering the intricate cellular processes within an inaccessible organ such as the brain, by bridging the gap between laboratory-based discoveries and real-life observations. **(A)** The intricate interplay between EVs and viruses *in vivo* operates on a multifaced level. EVs can serve as a mean of viral propagation or act as carriers of pro- or anti-viral mediators that might contribute to neurodegeneration or breakdown of the blood-brain-barrier (BBB). **(B)** Improved *in vitro* human models are warranted to delve into the role of EVs in the propagation of neurotropic viruses and to study EV dynamics. Such models might include organoid cultures or microfluidic systems mimicking the cellular composition and microenvironment of the CNS compared to traditional cell culture models. **(C)** The potential of EVs to cross biological barriers provides a mean by which brain-derived EVs in the periphery can be utilized as a relevant source of information. Indeed, the elucidation of key cellular mechanisms at play in the brain during viral infection could lead to the identification of robust biomarkers using the protein, nucleic acid, or lipid EV-associated signatures that are unique to viral infections. However, challenges such as sensitive isolation methods need to be addressed for standardized and efficient disease diagnosis and monitoring, thus ultimately improving patient care. Illustration created with BioRender.com.

The intricate relationship between EVs and viruses operates on multiple levels. EVs might serve as a vehicle of viral propagation. Moreover, transported viral or cellular content might evoke distinct responses in recipient cells (**Figure 1A**). As per virus point-of view, these responses can either be advantageous, priming cells for infection, or detrimental, hindering viral replication. Nevertheless, our comprehension of the role of EVs in viral infection, especially those pertaining to the CNS, remains incomplete, primarily because of the difficulty of isolating single cell specific EVs *in vivo*. While significant insights have been gained through cell culture models, the complexity of the CNS cellular composition and microenvironment is not fully captured yet. Similarly, while animal models offer valuable insights into EV dynamics in a physiological context, they may fail to mimic certain developmental or pathological features that are unique to humans, including specific human host-pathogen interactions (**Figure 1A**) (Bárbara and Sónia, 2017; Swearengen, 2018). To address these challenges, future research should focus on developing more physiologically relevant *in vitro* human models, such as organoid cultures or microfluidic systems, that better mimic the complexity of the CNS microenvironment (**Figure 1B**). The capacity of EVs to cross biological barriers, including the BBB, means that brain-derived EVs could be identified in the plasma. Characterization of brain-derived Evs in the blood might thus be used for diagnostic and prognostic assessment of various neurological disorders and real-time monitoring of patient responses to targeted therapies. However, current techniques for evaluating brain-derived EVs in CSF and blood face challenges due to the need for sensitive and specific isolation methods. Subsequently, there is an urgent need to standardize brain-derived EV isolation and identification methods to support efficient disease diagnosis (**Figure 1C**) (Sandau et al., 2024). Nevertheless, while research into brain-derived EVs in the context of virus-induced neurological diseases is still in its infancy, it holds significant promise as EVs 1) could serve as valuable sources of information that can help to elucidate intricate cellular processes within an inaccessible organ such as the brain and 2) hold promise for advancing clinical diagnoses.

## Author contributions

LO: Conceptualization, Writing – review & editing, Writing – original draft. RP: Writing – review & editing, Funding acquisition, Supervision. AM: Supervision, Writing – review & editing, Conceptualization, Investigation, Project administration, Visualization.
